# Automated segmentation of clinical CT scans of the cochlea and analysis of the cochlea's vertical profile

**DOI:** 10.1016/j.heliyon.2024.e35737

**Published:** 2024-08-06

**Authors:** Michael Siebrecht, Jeroen J. Briaire, Berit M. Verbist, Randy K. Kalkman, Johan H.M. Frijns

**Affiliations:** a Department of Otorhinolaryngology and Head & Neck Surgery, Leiden University Medical Center, PO Box 9600, 2300 RC, Leiden, the Netherlands; b Department of Radiology, Leiden University Medical Center, PO Box 9600, 2300 RC, Leiden, the Netherlands; c Leiden Institute for Brain and Cognition, PO Box 9600, 2300 RC, Leiden, the Netherlands

**Keywords:** Sensorineural hearing loss, Cochlear implant, Segmentation, Automated image analysis, CT, Cochlear anatomy

## Abstract

**Purpose:**

Knowledge of the cochlear anatomy in individual patients is helpful for improving electrode selection and placement during cochlear implantation, as well as in surgical planning. The aim of this study was to develop a model-free automated segmentation algorithm to obtain 3D surfaces from clinical computed tomography (CT) scans that describe an individual's cochlear anatomy and can be used to quantitatively analyze the cochlea's vertical trajectory.

**Methods:**

Clinical CT scans were re-oriented and re-sliced to obtain mid-modiolar slices. Using these slices, we segmented the cross-section of the cochlea.

**Results:**

3D surfaces were obtained for the first 1.5 turns of 648 cochleae. Validation of our algorithm against the manually segmented ground truth obtained from 8 micro-CT scans showed good agreement, with 90 % area overlap and an average distance of 0.11 mm between the segmentation contours. The average cochlear duct length for the basal turn was 16.1 mm along the central path and 22.4 mm along the outer wall. The use of an intrinsic, observer-independent coordinate system and principal component analysis enabled unambiguous quantitative evaluation of the vertical trajectory of the cochlea, revealing only a weak correlation between the symmetry of the commonly used basal turn diameters (B-ratio of A and B diameters) and the profile of the vertical trajectory.

**Conclusion:**

A model-free segmentation algorithm can achieve similar accuracy as previously published methods relying on statistical shapes. Quantitative analysis of the vertical trajectory can replace the categorization into rollercoaster, sloping, or intermediate vertical trajectory types.

## 1 Introduction

The human cochlea roughly follows the shape of a logarithmic spiral, with approximately 2.5 turns. In the last few decades, the majority of anatomical studies of the human cochlea were performed to obtain shape and size information for cochlear implantation [[1], [2], [3], [4], [5]]. In the present study, we describe an algorithm designed to obtain a 3D surface describing an individual human cochlea, which could be a foundation for measuring cochlear dimensions with high accuracy.

The availability of patient-specific information on the cochlear anatomy would allow personalized cochlear implantation in terms of both electrode array choice and the insertion method during surgery [6]. Complications, such as translocation from the scala tympani to the scala vestibuli, incomplete insertion, or electrode array tip fold-over, during cochlear implantation surgery have decreased in recent years, though they still occur [7,8]. Knowledge about the patient's cochlear anatomy could identify which patients are at risk of such complications and potentially help decrease them further [1,4,9].

A large dataset of anatomical descriptions of cochleae would also allow a robust study of the variability in human cochlear anatomy. Past studies on this variability can be divided into either in vitro studies using histological sections or micro computed tomography (CT) scans, or in vivo studies based on clinical CT scans. In vitro micro-CT studies allow for the analysis of great anatomical detail and have shed light on the principal variations in cochlear shape and size [1,9]. However, a disadvantage of these studies has been the relatively small sample sizes. Moreover, due to the large radiation dose, small specimen size, and long acquisition time, micro-CT scans are not applicable to living subjects.

In contrast, clinical CT scans of the cochlea are obtained as part of the clinical routine in many hospitals before cochlear implantation and, therefore, are available in much greater numbers. Many studies have investigated these clinical CT scans by measuring prominent features, such as the overall cochlear diameter, or sampled points along the outer and inner walls of the cochlea [2,5,10,11]. Another approach has been to fit parametric models to the cochlea [12]. Yet other studies have used a shape model built from micro-CT scans of either the scala tympani alone [13] or the complete cochlear lumen [[14], [15], [16]] to segment a cochlea imaged using a clinical CT scan. However, the number of micro-CT scans used to build these shape models may be too small to capture the complete variability of the anatomy. Recently, artificial intelligence has been employed to segment the cochlea through deep learning [17].

Our research group previously published an automated tracing method that relied solely on clinical CT scans for tracing the outer, inner, and bottom walls of the cochlea [4]. Though many measurements can be made using these coordinates, they do not provide all measures of interest for cochlear implantation. Some crucial measurements rely on re-orientation of the cochlea, such as the vertical ascending profile of the cochlea [18,19]. Such re-orientation requires data on the complete cochlear surface.

To perform a correlation study between anatomy and implantation outcome, a large sample of individual cochlear morphologies is needed to achieve sufficient statistical power. Manually extracting the 3D surface of the cochlea from clinical CT scans would be an error-prone, cumbersome, and time-consuming process. Therefore, an automated method of segmenting the cochlea is needed to obtain individual measurements in a large dataset.

Segmentation is the separation of an image or image volume into a background and one or multiple objects. These segmented morphologies are more versatile than discrete measurements. Based on the success of the tracing algorithm reported by Van der Jagt et al. [4], we decided to extend and modify it into an algorithm for complete segmentation of the cochlea. Considering the range of quality of CT scans in our dataset, the new algorithm was designed to be as robust as possible in order to include as many patients as possible. To achieve this robustness, the segmentation was confined to the first 1.5 turns of the cochlea, covering the entire length of most of the cochlear implant electrode array designs in current use. For validation purposes, automated segmentation of low-resolution CT scans was compared with manual segmentation of high-resolution micro-CT scans, and measurements of the cochlear duct length through the first cochlear turn were checked against previously published results [20].

Another aspect of cochlear anatomy that is of interest for cochlear implantation is the vertical profile. Large changes in the slope of the vertical profile have been hypothesized to pose an increased risk of trauma during electrode array insertion [1,4,9]. Demarcy et al. [18] found a pronounced bimodal distribution of the vertical profile. They investigated the parameter that controls the bumpiness of the vertical profile in their model and showed that the distribution had two peaks. Pietsch et al. [21] suggested a link between a newly introduced B-ratio (semi-minor axis of the basal turn divided by cochlear width) and the incidence of rollercoaster-type profiles.

The first aim of the present study was to develop a new algorithm for the segmentation of clinical CT scans and validate that algorithm using micro-CT scan data. The second aim was to revisit the bimodal distribution of the vertical profile and test the suggested qualitative link between the B-ratio and the incidence of rollercoaster-type profiles using quantitative principal component analysis (PCA).

## 2 Materials and methods

### 2.1 CT scans

As part of the clinical routine in our hospital, all cochlear implant candidates undergo a pre-operative CT scan using an Acquilion scanner (Toshiba Medical Systems, Otowara, Japan) with an isotropic voxel size of 0.3 mm (rotation time 1.5 s, tube voltage 120 kV, tube current 100 mA, and reconstruction algorithm FC81).

### 2.2 Design of the segmentation algorithm

**Fig. 1:**
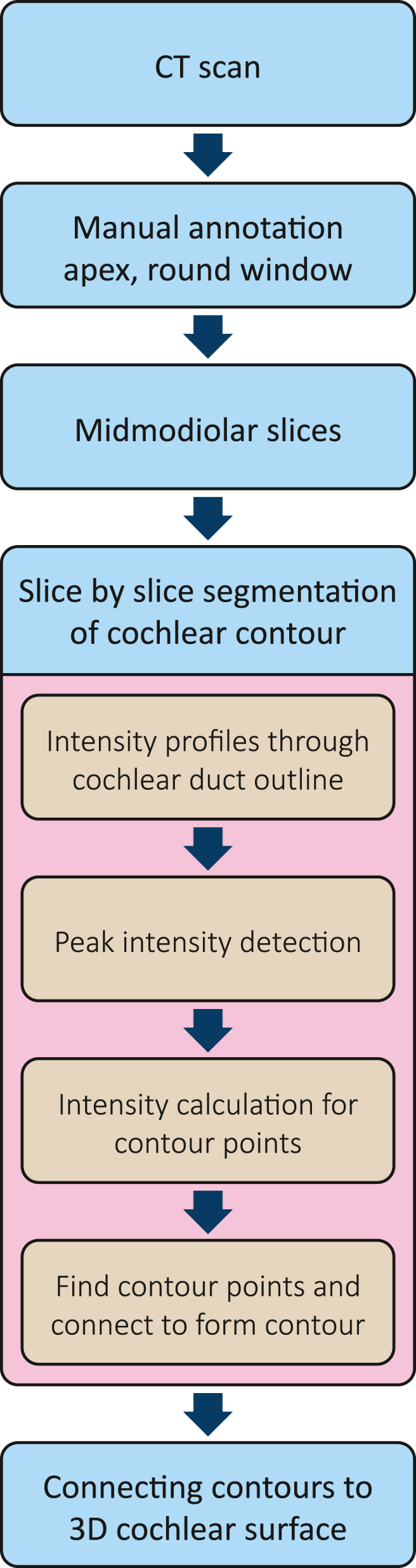
Flow diagram of the segmentation algorithm.

**Fig. 2:**
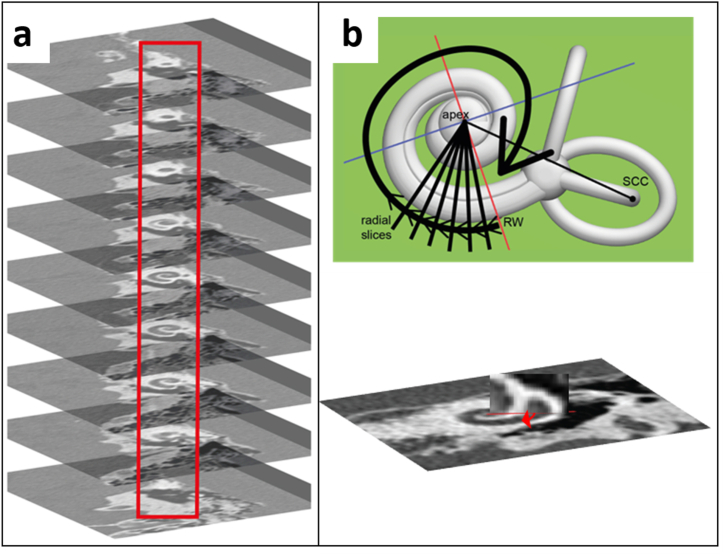
Re-slicing of the CT dataset. a, Slices were parallel to the basal turn of the cochlea. b, Radial mid-modiolar re-slicing with the modiolus as the central axis, starting at the round window (RW) and perpendicular to the planar slices.

We designed an automated algorithm to segment the cochlea on a clinical CT scan, the general process of which is outlined in Fig. 1. This design expanded upon a cochlear wall tracing algorithm previously developed at our institution [4]. The method requires manual pre-processing of the clinical CT scan to re-orientate and obtain mid-modiolar slices. This manual step was followed by a re-slicing of the CT scan into radial mid-modiolar slices containing approximately orthogonal cross-sections of the cochlear duct (Fig. 2). Next, the contours of the cochlear duct were determined in each mid-modiolar slice and connected to obtain a 3D surface describing the cochlear duct.

#### 2.2.1 Manual pre-processing

Multiplanar reconstructions were made from the clinical CT scan volume using Vitrea software (Vital Images, Minnesota, USA). The in-plane resolution of the multiplanar reconstructions was set to approximately 0.07 × 0.07 mm using bicubic interpolation. The orientation of these multiplanar reconstructions was parallel to the basal turn of the cochlea in accordance with the consensus cochlear coordinate system [22]. Subsequently, the apex and round window (RW) were annotated manually.

#### 2.2.2 Automated segmentation

The algorithm for automated segmentation considers two different sets of angles: the cochlear angle and the circumferential angle. The cochlear angle was defined as the angle between the location of the cross-section and the RW, or the ‘degrees (°) from the RW’.

#### 2.2.3 Initialization

The multiplanar reconstruction (Fig. 2a) was re-sliced into radial mid-modiolar slices in 1° steps with the RW as the angular starting point (Fig. 2b). See van der Jagt et al. [4] for details. The z-direction resolution (vertical) was also up-sampled using bicubic interpolation to a pixel spacing of approximately 0.07 mm to achieve an isotropic pixel size for the mid-modiolar slices. Integer pixel values were used as values for distance-related parameters in our algorithm to allow for direct image indexing.

Next, the segmentation routine was initialized by estimating the center of the cochlear duct cross-section approximately 30° from the RW. Following initial tests, this center location was estimated by setting its radial distance at 72 % of the radial distance between the RW and the modiolar axis (z-axis) and setting its height 1 mm above the manually selected RW location. This positioned the center inside the cochlear duct for all 669 CT scans that were analyzed.

#### 2.2.4 Slice-by-slice segmentation

**Fig. 3:**
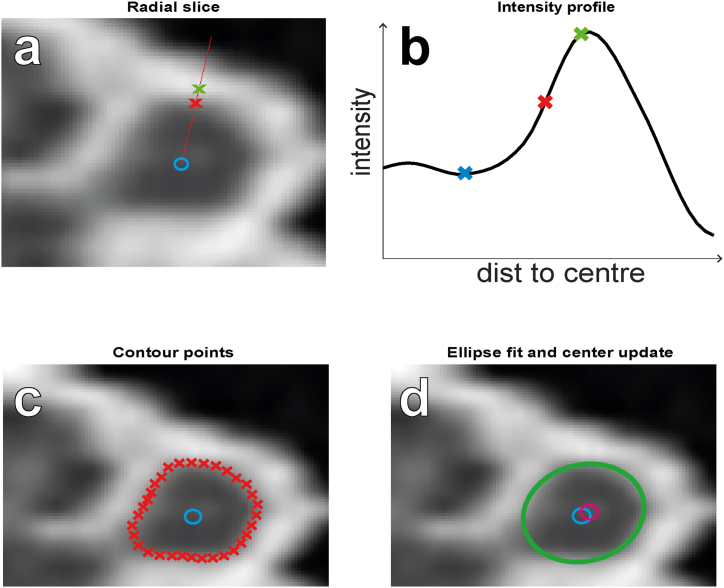
Slice-by-slice segmentation. a, Radial slice showing the estimated center of the cochlear duct cross-section (blue circle) and one exemplary ray (red line), as well as the detected peak (green cross) and resulting contour point (red cross). b, Intensity profile along the exemplary ray, showing the detected peak (green cross), valley (blue cross), and resulting contour point (red cross). c, The contour points for all rays (red crosses). d, The ellipse (green contour) fitted to the contour points and the resulting center shift from the old center position (blue circle) to the new center position (magenta circle). (For interpretation of the references to colour in this figure legend, the reader is referred to the Web version of this article.)

The algorithm determined the contours of the cochlear duct in each mid-modiolar slice. Fig. 3 illustrates the process for finding the contour 30° from the RW. The method consists of casting a ‘ray’ in the mid-modiolar slice starting at the previously estimated center of the cochlear duct and pointing in an arbitrary direction (Fig. 3a). An ‘intensity profile’ was calculated along this ray by interpolating the voxel values in the 3D CT stack. These values were given in Hounsfield units (HU), which will be referred to as ‘intensities’ and generally indicate material density, with bone having high intensity and the interior of the cochlear duct, which largely consists of perilymph, having low intensity.

The algorithm then determines peaks and valleys in the intensity profile (Fig. 3b), with the assumption that the edge of the cochlear duct will be located between an adjacent valley and peak. If there were multiple peaks along the ray, the appropriate one was selected in a later stage of the algorithm.

Van der Jagt et al. [4] assumed that the intensity values at the cochlear walls (or contour intensity) are halfway between the intensity values of the nearest valley and peak. However, due to partial volume effects, the thickness of a bony boundary affects its image intensity on the CT scan. In an idealized 2D situation, in which the bone is a 2D rectangle, there are three cases to consider. If the bone layer extends beyond 1 voxel, then the intensity at the center of the bone corresponds to 100 % of the true bone density, and the intensity at the edge of the bone corresponds to the 50 % point between bone and fluid density. If the thickness is 0.5 voxels, then the intensity at the center of the bone corresponds to the 50 % point, and the intensity at the edge of the bone also corresponds to the 50 % point. This is equivalent to a thickness of <0.5 voxels. For a thickness of 0.5–1 voxels, the relative intensity between the bone boundary and bone core is between the two extremes. The real-world situation is more complicated than this thought experiment given the curved 3D anatomy of the cochlea and effects that the scanning procedure and reconstruction algorithm might have on the exact averaging of true tissue densities within a voxel. Therefore, the thresholds used for our algorithm were determined experimentally.

In the refined version of the cochlear wall detection algorithm, the halfway assumption is only followed if the peak intensity is > 1000 HU. If the peak intensity is < 300 HU, the contour intensity is assumed to equal the peak intensity. If the peak intensity is between 300 and 1000 HU, the contour intensity is assumed to be positioned proportionally between the peak intensity and the average of the minimum and peak intensity.

The algorithm calculated intensity profiles along 32 rays with lengths of 40 pixels (i.e., approximately 2.8 mm) pointed in all directions at equal angular spacing from each other (i.e., 11.25°). Contour points were determined along each ray in the manner described above; if multiple contour points were detected for a ray, the algorithm chose the one that was closest to the contour point from the previous slice for the ray with the same orientation (Fig. 3c).

To obtain the new center position as the starting point for the next slice, an ellipse was fitted to all eligible contour points (Fig. 3d). The center of the ellipse was defined as the central point between both focal points of the ellipse (i.e., the center of mass of the ellipse). The center position of the cochlear duct was then updated as the average ellipse center of the last 10 slices.

Next, the eligibility of the contour points for all rays was checked. For the contour points along all rays, the distance to both focal points of the ellipse was summed. All contour points for which this summed distance deviated from the average summed distance by more than 5 pixels were rejected. This eligibility criterion was implemented to deal with exceptions in the appearance of the bony capsule around the cochlear lumen, such as in the region where the facial nerve is in close proximity to the cochlea.

Finally, the algorithm advanced to the next slice by a 1° step and repeated the segmentation procedure as described above until it reached 540° from the RW, or 1.5 turns of the cochlear spiral, where it terminated. Following this forward run, a backward run was then performed from 40° after the RW to 10° before the RW as described above, except the center position and the contour points that were established during the forward run were used as a starting point. The backward run both corrected for segmentation errors caused by poor initialization of the forward run and filled in the region around the RW that was skipped initially.

### 2.3 Segmentation accuracy

**Fig. 4:**
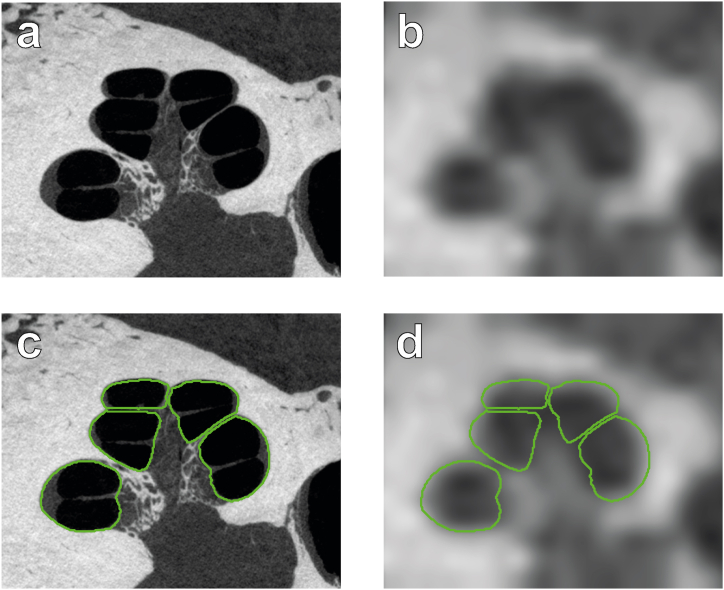
Illustration of the degrading of the micro-CT data, and the ground truth segmentation. a, Mid-modiolar slice of the high-resolution micro-CT scan. b, Degraded version of the micro-CT scan (compare Fig. 5 and Fig. 7 for clinical CT scans). c, Ground truth segmentation overlay (green contours) on the micro-CT. d, The degraded clinical-like version of the micro-CT scan with the ground truth overlay (green contours). (For interpretation of the references to colour in this figure legend, the reader is referred to the Web version of this article.)

**Fig. 5:**
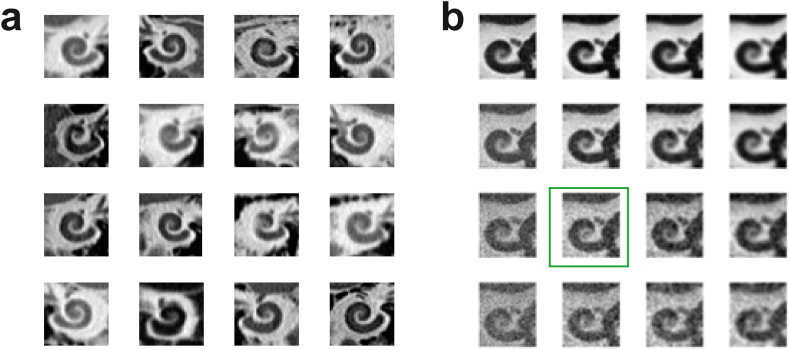
Matching the quality of the clinical CT images by degrading micro-CT scans. a, Range of clinical CT quality. b, Different levels of micro-CT degradation, from which the best match to clinical CT was chosen (green box). (For interpretation of the references to colour in this figure legend, the reader is referred to the Web version of this article.)

The ground truth was obtained by manual segmentation of micro-CT scans of the temporal bone (Fig. 4a and c). Micro-CT scans allow for straightforward manual delineation of the cochlea; in contrast, even modern state-of-the-art clinical CT scans do not offer enough resolution and contrast to manually delineate the inner or upper boundary from the second cochlear turn upwards. Rather than obtaining another scan of the same temporal bone with a clinical scanner, the high-resolution micro-CT was degraded to match the image quality of an in vivo clinical CT scan. A combination of blurring and additive noise was used for the degradation, similar to standard methods in, for example, no-reference image quality assessment (NR-IQA) research [23]. First, the image resolution was matched to that of typical clinical CT scans (Fig. 5a). Next, the image stack was blurred with a 3D Gaussian filter 1.48-pixels wide, 30 % noise from a uniform distribution was added, and the image stack was blurred again with a 3D Gaussian filter 2.92-pixels wide. These values for blur and noise were selected by having a radiologist with extensive experience with head and neck scans inspect a series of images that were degraded with a range of blur and noise and picking the one that most closely resembled clinical CTs (see Fig. 5b).

Segmentation was achieved for the degraded dataset using our automated segmentation algorithm (Fig. 4b and d). The accuracy of the automated segmentation was assessed using two quantitative measurements: the Dice ratio, which measures the overlap between the area of the automated segmentation and the ground truth [24], and the modified Hausdorff distance measure, which provides the distance between the contours of the automated segmentation and ground truth [25]. To track the performance of our algorithm for the different parts of the cochlea, error measures were calculated at each cochlear angle. This allowed us to assess the expected decrease in accuracy as the cochlear spiral tightened. We also divided the contour into four quadrants (upper, lower, inner, and outer boundaries of the cochlear duct) to obtain individual distance measures. This allowed independent monitoring of the accuracy for the highly visible outer boundary of the cochlear lumen compared to the poorly visible inner boundary.

### 2.4 Measuring cochlear length and PCA of the vertical cochlear profile

**Fig. 6:**
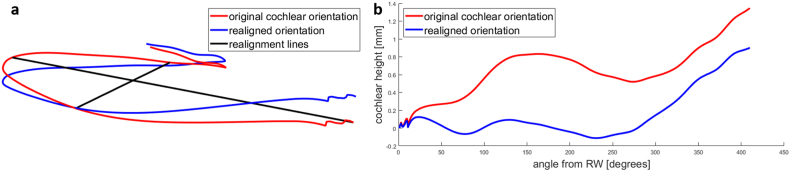
3D plots showing the trajectory of the center of the cochlear duct as determined by the automatic segmentation algorithm in the initial and re-aligned forms. a, Re-orientation of the cochlea to an unambiguous, intrinsic coordinate system defined by two lines through the basal turn of the cochlea. One line runs from the round window across the cochlea to the 180° point, and the second line runs perpendicular to the first line from the 90°–270° points. b, Vertical profiles determined with both the original and realigned orientation. Note the flat trajectory of the basal turn in the re-oriented cochlea, revealing its planar shape, which is obfuscated in the original coordinate system.

All cochleae were realigned to a uniquely defined intrinsic coordinate system, which was a plane defined by two lines (Fig. 6a). The realignment was based on the trajectory of the center of the cochlear duct determined by our segmentation algorithm, as this is defined intrinsically. To increase the robustness of the central cochlear duct trajectory, we smoothed the center points with a zero-phase running average filter of 40°.

The first line used to realign the coordinate system was drawn through the center of the RW and the center of the cochlear duct 180° from the RW. The second realignment line was set perpendicular to the first line, between the centers of the cochlear duct at 90° and 270°. This realignment removed any coordinate system bias introduced by the manual preprocessing of the CT scans. The realignment method was tailored to a cochlear segmentation of the most commonly implanted section of the cochlea, which is the most basal 1 to 1.5 turns of the cochlea. In contrast, the method used by Demarcy et al. [18] would have required all turns of the cochlea.

The vertical profiles were extracted from these realigned cochleae (Fig. 6b). PCA was performed to reduce the dimensionality of the vertical profiles. We checked the distribution of the eigenvalues for signs of bimodality that would support the hypothesis of distinct vertical profile categories as suggested by the bimodal distribution found by Demarcy et al. and the rollercoaster, intermediate, and sloping categories reported by Avci et al. [1].

The B-ratio was calculated and the distribution compared to that reported by Pietsch et al. [21] to check whether our segmentation routine or the reorientation of the cochlea strongly altered the measurement. Finally, the lengths along the center of the cochlear duct and along the outer wall were calculated. In this way, these results could be compared with the results from other studies, which served as further validation of our segmentation algorithm.

## 3 Results

### 3.1 Robustness of the algorithm

We analyzed 669 CT scans and obtained the 3D surface of the cochlear lumen for the first 1.5 turns on 648 scans (97 %). For the remaining 21 scans, the algorithm terminated prematurely, ranging from an immediate failure to termination just prior to achieving 1.5 turns. In most cases, this was due to too many ineligible contour points at the top boundary of the second turn due to the vanishing contrast between the second and third turns.

### 3.2 Accuracy of segmentation based on visual inspection

**Fig. 7:**
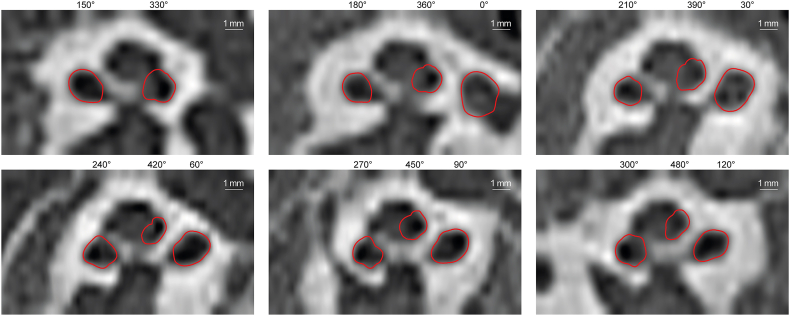
Overlay of segmented cochlear contours on clinical CT scans from the round window to 480° in 30° steps. The segmentation was successful with expected deviations in the second turn, especially at the inner and top walls where visual validation is challenging.

Visual inspection of the overlay of the segmented contours had promising results. However, the boundary between the second and third turns was very difficult to differentiate visually because, with a thickness <0.04 mm (as measured on a micro-CT scan), it is very thin compared to the 0.3 mm resolution of clinical CT scans (Fig. 7).

### 3.3 Validation against ground truth from micro-CT scans

**Fig. 8:**
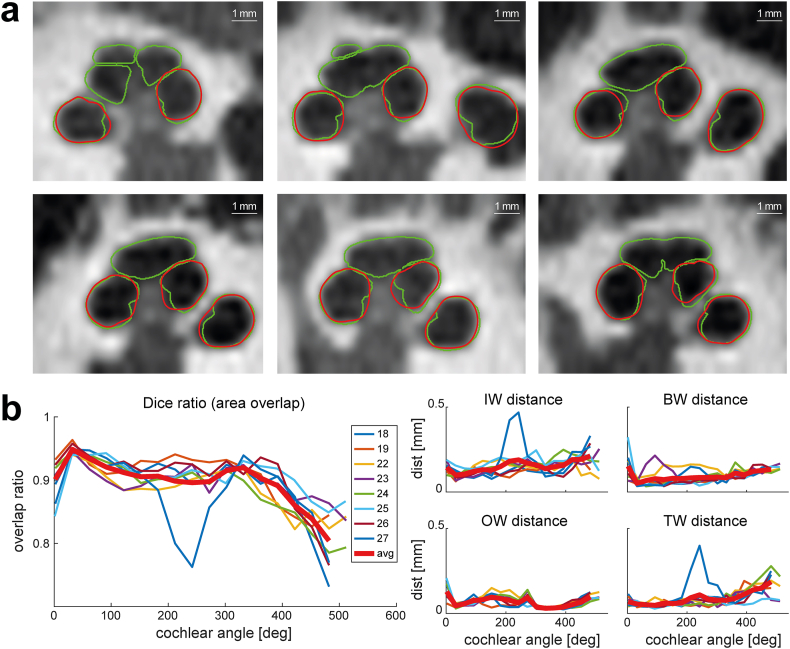
Validation of the segmentation method. a, Visual comparison of our automated segmentation result from the full resolution dataset (i.e., ground truth; green contours) and the degraded dataset (red contours), showing good agreement but slightly larger errors for the inner wall, especially in the second turn. b, Quantitative measurements of the segmentation accuracy in terms of area overlap using the Dice ratio, and distance between the segmentation contours using the modified Hausdorff distance. The contours are split into four parts to separately calculate the modified Hausdorff distance for the inner wall (IW), outer wall (OW), bottom wall (BW), and top wall (TW). (For interpretation of the references to colour in this figure legend, the reader is referred to the Web version of this article.)

The algorithm was validated against the ground truth based on eight high-quality micro-CT scans. A visual comparison showed a high degree of agreement between the contours for the first turn (Fig. 8a). Starting in the second turn, the algorithm often failed to detect the boundary between the second and third turns correctly, and the inner boundary of the second turn could be segmented less accurately. These qualitative observations were confirmed by the objective measures. For the first turn, the overall accuracy of the segmentation was 90 % in terms of the Dice ratio, the overlap between the segmented area of the ground truth, and our algorithm; the overall distance error was 0.11 mm. From the second turn onwards, the Dice ratio decreased gradually but still maintained an average 80 % accuracy at 500°. Due to the decline in accuracy beyond 400°, the vertical profiles were only measured up to this point. Looking at the four cross-sectional quadrants of the contour, the algorithm struggled with the top boundary of the second turn. The error remained <0.08 mm for the basal turn but increased to 0.22 mm at 500°. The algorithm also struggled with the inner boundary, starting with an error of 0.09 mm at 45° and increasing to an error of 0.3 mm at 500°. The algorithm had a higher accuracy for the lower quadrant, with the error increasing from 0.07 mm at 45° to its largest value of 0.16 mm at 500°; the highest accuracy was seen for the outer boundaries, with the error remaining <0.11 mm independent of the cochlear angle (Fig. 8b).

### 3.4 Cochlear length

**Fig. 9:**
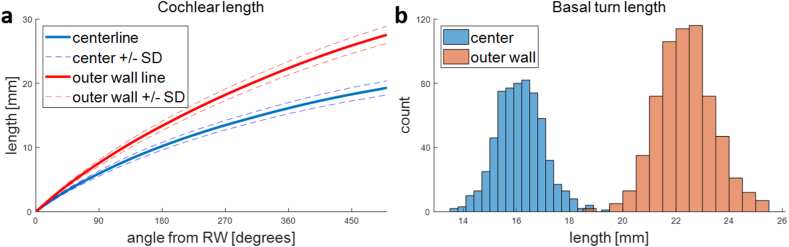
Quantitative results of cochlear lengths. a, Cochlear length along the outer wall and center line. b, Distribution of basal turn length measured along the outer wall and center line.

The lengths of the cochlear ducts along the outer wall and along the center of the ducts were measured starting from the RW to a cochlear angle of 500° (Fig. 9a). The basal turn length (BTL) is a common measurement, both along the outer wall and at the center of the cochlear duct. The average length of the first turn along the outer wall was 22.4 mm, with a standard deviation (SD) of 1.1 mm (Fig. 9b). The length along the center of the cochlea was 16.1 mm (SD 0.9 mm) on average (Fig. 9b).

### 3.5 Rollercoaster-type vertical profiles

**Fig. 10:**
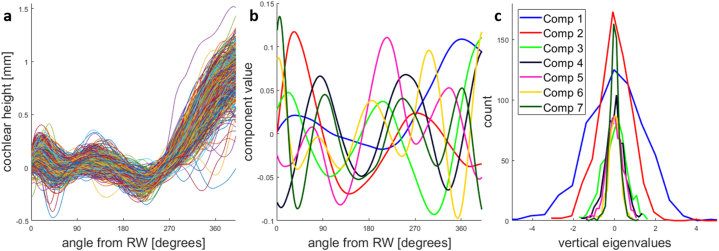
Principal component analysis of the vertical cochlear profiles of 648 patients. a, All realigned vertical profiles. The basal turn is flattened by the realignment. b, The first seven principal components (Comp), capturing 95 % of the variability of the vertical profiles. The first component mainly captures the total height of the cochlea, and the remaining components capture by how much and at what cochlear angle the vertical profiles deviate from a monotonically increasing slope. c, The monomodal distributions of the eigenvalues for the first seven principal components show that all features of the vertical profiles are expressed as a continuum and do not form distinct categories.

Fig. 10a shows all 648 vertical profiles of the realigned cochleae, with the results of the PCA in Fig. 10b and c. The first seven components account for more than 95 % of the variability; therefore, only these seven components were used in further analyses. The distribution of all coefficients was roughly normal and had a single peak.

**Fig. 11:**
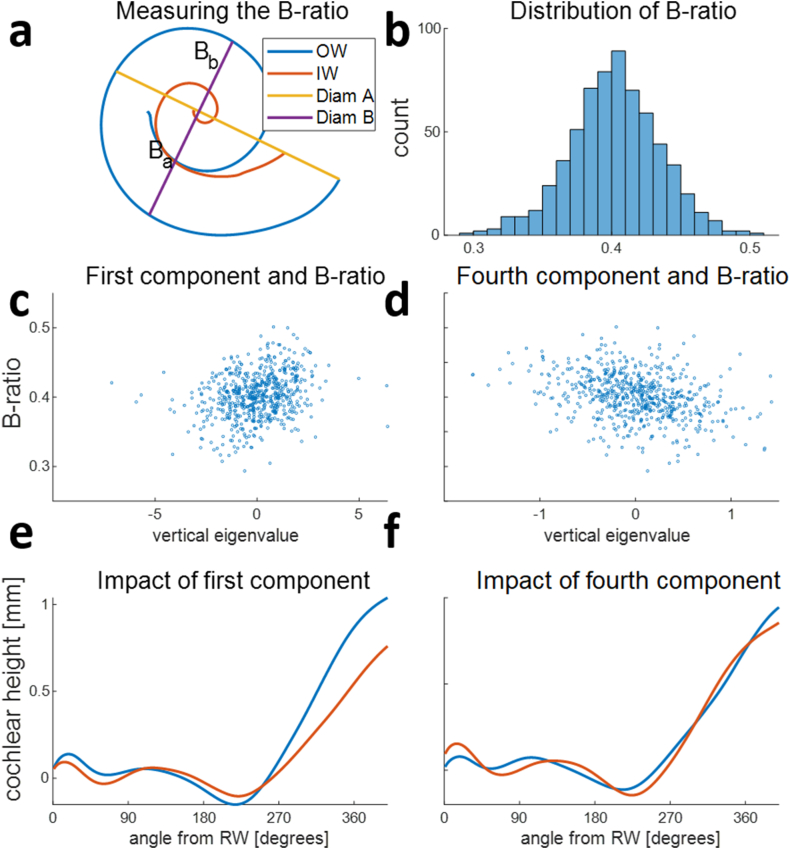
Correlation between the B-ratio and principal components of the vertical cochlear profile. a, Cochlear outer wall (OW), inner wall (IW), and the measurements of the B-ratio [Bb/(Ba + Bb)] as described in Ref. [21]. B, Variability of the B-ratio in 648 patients, showing a normal distribution (mean = 0.4, SD = 0.03). c-d, Scatterplots of the B-ratio and the principal components that significantly correlated with the B-ratio. c, A weak positive correlation (r = 0.32). d, A weak negative correlation (r = −0.26) e-f, The influence of the principal components on the vertical profile shape is shown as the average vertical profile + the component eigenvector (blue) and as the average vertical profile – the component eigenvector (red). e, The first component influences the bump height around 100° from the RW, as well as the total height at 400°. f, The impact of the fourth component is more subtle and mainly consists of a shift of the bump between 100° from the RW to 150° from the RW. (For interpretation of the references to colour in this figure legend, the reader is referred to the Web version of this article.)

Fig. 11 shows the correlation analysis between the B-ratio and the principal components of the vertical cochlear profile. The B-ratio, as defined by Pietsch et al. [21], measures the asymmetry of the basal turn (illustrated in Fig. 11a). Our 648 B-ratio measurements were normally distributed around an average B-ratio of 0.4 (Fig. 11b). The correlation between the B-ratio and vertical profile coefficients was assessed to test the hypothesized connection between the B-ratio and the degree to which the vertical profile corresponded to a rollercoaster profile.

Two of the first seven principal components correlated significantly with the B-ratio (Fig. 11c and d). However, this correlation was weak (first component, r = 0.32; fourth component, r = −0.26). The first component influenced the height of the ‘bump’ around 100° from the RW, as well as the total height at 400° (Fig. 11e). The impact of the fourth component was more subtle, mainly consisting of a shift of the bump location from 100° to 150° (Fig. 11f).

## 4 Discussion

We have presented a novel automated method for segmentation of the human cochlea on clinical CT scans and generated the largest sample of cochlear segmentations to date. For the segmentation algorithm, we extended and modified the idea behind the Van der Jagt algorithm [4]. In contrast to the Van der Jagt algorithm, which traces the inner, outer, and bottom walls of the cochlea, the new algorithm segments the entire cochlear duct. In addition to multiplanar reconstruction of the CT volume, the approximate RW and apex positions have to be located manually, but our automated segmentation algorithm requires no specialized radiological training. The segmentation method itself requires no manual interaction.

For an unbiased analysis of the vertical cochlear profile, we developed a robust and intrinsic coordinate system. We introduced image interpolation between the slices of the multiplanar reconstruction stack to improve precision in the z-dimension. In addition, moving the origin of the intensity profiles from the modiolus to the center of each cochlear duct cross-section allowed a dynamic contour level to replace the fixed 50 % approximation of the Van der Jagt algorithm. This particularly helps in regions where the otic capsule is thinner, such as in the region where the facial nerve runs past the cochlea.

We compared the performance of the new algorithm with that of the Van der Jagt algorithm by taking the inner, outer, and bottom cochlear duct boundaries determined by the Van der Jagt algorithm and comparing them to their equivalent points in the contours determined by the new algorithm at each cochlear angle. The average mismatch between the two algorithms was less than 1 voxel on the CT scan. For the outer wall, the mismatch was 0.06 mm (SD ± 0.03 mm) on average. Visual inspection of the larger mismatches (0.1–0.2 mm) revealed a comparable number of errors for both methods; in some cases, it was not possible to determine which method had performed better even for these larger mismatches. For the new algorithm, larger errors mainly occurred near the RW, whereas the Van der Jagt algorithm showed larger errors associated with the facial nerve region. The average mismatches for the inner and bottom walls were larger, 0.18 (±0.07) mm and 0.22 (±0.06) mm, respectively. This was expected due to the much weaker contrast compared to the outer wall and, therefore, the higher level of uncertainty that the algorithms deal with. This poor contrast also prevented visual inspection to determine which algorithm was correct in the case of a mismatch.

Noble et al. [13] and Kjer et al. [14] previously reported automated methods for segmentation of the cochlear anatomy. Both used active shape models to learn the anatomical variation from a micro-CT dataset and then registered the learned shape model to clinical CT scans. However, Kjer et al. used a larger training set and volume to volume registration, whereas Noble et al. used mesh to volume registration. Both of these methods segment the whole cochlea, whereas our method focused on the region of the cochlea relevant for electrode arrays in cochlear implantation. In addition, Kjer et al. and Noble et al. separately segmented the scala vestibuli and scala tympani, whereas we segmented the whole cochlear duct.

Similar to Kjer et al. and Noble et al., we validated our algorithm against a micro-CT ground truth. Although these studies ran their automated segmentation on clinical CT scans of the same temporal bones from which the micro-CT scans were taken, we ran our automated segmentation on a degraded version of our micro-CT scans because we did not have access to the temporal bones for re-scanning using a clinical scanner. However, we could match the quality to that of clinical CT scans of living human patients. Clinical CT scans of isolated temporal bone specimens have better quality but can underestimate the errors that automated segmentation may make when scanning a real patient. One other important difference between our algorithm and previously published algorithms is that it only covers the first 1.5 cochlear turns, rather than attempting to segment the entire cochlea due to the fact that, in clinical CT scans, the different turns of the cochlear duct and the modiolus cannot generally be distinguished from each other in the cochlear apex, which means that segmenting the apical section of the cochlea requires the use of ‘extrapolating methods’, such as statistical shape models or machine learning. However, it should be noted that the first 1.5 cochlear turns cover the insertion depth of most currently implanted electrode arrays [26]. Due to their differences, comparisons of our method to that of Kjer et al. or Noble et al. should be made with caution. Though the segmentation task of our method can be argued to be simpler, it achieves the same accuracy as other state-of-the-art methods in the field. We are confident that our detailed analysis of accuracy, by both angle and cross-sectional quadrant, provides valuable information for interpreting the segmentation results in future applications.

Previously published methods of determining the length of the cochlear duct were either based on variations of the parameter method by Escudé et al. [2,5,27], or by using statistical shape models [[13], [14], [15], [16]]. The method presented here showed that direct measurements of cochlear duct length for the basal turn of the cochlea were feasible, and that their results were within the previously reported range [1,[28], [29], [30]], both for measurements along the outer wall and for the measurement along the central path. All four comparisons could be made for the BTL measured along the outer wall, but only one study has also measured the length along the central path [1]. This agreement corroborates the accuracy of our algorithm. Our measurement is, on average, a bit larger than the previously reported range for the BTL, which may be due to manual assessments underestimating the size of the cochlea in general [31].

The course of the vertical trajectory of the cochlea has been hypothesized to affect cochlear implant insertion. A trajectory resembling a rollercoaster has been thought to carry an increased risk of intracochlear damage during surgery [1,4,9]. As illustrated in Fig. 6b and shown by Demarcy et al. [18], a small rotation of the central axis can change the vertical profile of the cochlea dramatically. Checking our large dataset of cochleae, we found a significant inter-observer difference between the vertical profiles, despite the two researchers trying to achieve the same cochlear coordinate system. For this reason, we chose to realign the cochleae to an unambiguous, intrinsic coordinate system.

Using a realignment method that is intrinsic, meaning that it does not depend on manual pre-processing steps or annotations (e.g., the definition of the modiolar axis), can remove bias introduced during multiplanar reconstruction. In other words, it is independent of the observer. The method previously published by Demarcy et al. [18] could not be applied to our segmentations because it requires complete segmentation of the cochlea up to the apex. Therefore, we used a different intrinsic method that could be applied to segmentation of the first 1.5 turns. Our method is very easy to implement, requiring only four points along the central trajectory of the cochlear duct.

The availability of CT segmentation methods has led to the creation of clinically applicable segmentation software modules, like Otoplan (MED-EL [32,33]) and CImago (Advanced Bionics [15,16]). These tools produce fully automatic segmentation and detailed information on the cochlea and analysis of possible trauma due to the insertion. The drawback of these applications is, however, that they are produced by the CI manufacturers and are only useable for their specific implants.

Pietsch et al. [21] manually analyzed the relationship between the B-ratio and the shape of the vertical profile. Using visual inspection, they observed more rollercoaster-type profiles in the group with a smaller B-ratio. We quantitatively tested their finding using PCA to characterize vertical profile shapes. Though the correlation between the shape of the vertical profile and the B-ratio confirmed their finding in principle, the correlation was weak. One reason why the strong association they reported was not found by our analysis may be that the symmetry of the basal turn and the vertical trajectory both depend on the manually defined coordinate system. Tilting the cochlea out of the basal turn plane will simultaneously increase asymmetry and create additional up and down slopes in the vertical profile. Our realignment of the profiles makes the coordinate system observer-independent and, thus, overcomes this issue. We also performed PCA on the original vertical profiles and observed an even weaker correlation with the B-ratio. This may be due to a difference between our manual coordinate system and Pietsch et al.’s coordinate system, which can be seen when comparing our representative vertical profile before realignment (Fig. 6) with the general shape of their vertical profiles. However, our different segmentation method and the realignment did not have a strong effect on the B-ratio measurement itself, as it had a very similar distribution to the one determined by Pietsch et al. [21].

Demarcy et al. [18] suggested a pronounced bimodal distribution of vertical profiles. None of our principal components exhibited a bimodal distribution, suggesting that their finding may be an artefact of their parametric fitting approach.

Our algorithm for the automated segmentation of the cochlea from clinical temporal bone CT scans provides patient-specific information on cochlear morphology. Combined with postoperative CT scans, the algorithm makes it possible to investigate large numbers of intra-cochlear positions and analyze the impact of the electrode array's position on speech understanding by recipients of cochlear implants. In turn, insights gained from these studies allow us to optimize the performance for future patients by personalizing cochlear implantation in terms of both implant choice and individually adjusted insertion.

In summary, we designed and validated an algorithm to obtain sub-resolution segmentation of the cochlea from clinical CT scans, which allows detailed analysis of an individual's cochlear anatomy in the context of cochlear implantation. We applied the algorithm to obtain a large dataset of cochlear morphologies and refined previous findings regarding the vertical profile of the cochlea, for which we developed a robust, intrinsic, and unbiased coordinate system. In contrast to previous studies, we did not find any evidence of a bimodal distribution of vertical cochlear profile types, and we demonstrated only a weak correlation between the B-ratio and vertical cochlear profile.

## Ethics statement

This study was reviewed and deemed exempt from ethics approval by The Medical Ethics Committee Leiden The Hague Delft with the reference number G17.002, dated October 9th, 2017.

Informed consent was not required for this study because the paper contains no identifying patient data; according to national law at the study location, no ethics approval or informed consent is needed for retrospective studies involving data from humans.

## Data availability

The imaging data used in this study is confidential clinical data and not openly available.

## CRediT authorship contribution statement

**Michael Siebrecht:** Writing – original draft, Visualization, Validation, Software, Methodology, Investigation, Formal analysis. **Jeroen J. Briaire:** Writing – review & editing, Supervision, Conceptualization. **Berit M. Verbist:** Supervision, Resources, Methodology, Data curation. **Randy K. Kalkman:** Writing – review & editing, Software. **Johan H.M. Frijns:** Writing – review & editing, Supervision, Funding acquisition, Conceptualization.

## Declaration of competing interest

The authors declare the following financial interests/personal relationships which may be considered as potential competing interests: Michael Siebrecht reports financial support was provided by Advanced Bionics LLC. If there are other authors, they declare that they have no known competing financial interests or personal relationships that could have appeared to influence the work reported in this paper.
